# Reducing use of desflurane in the anaesthetic department: A controlled interrupted time series analysis

**DOI:** 10.1177/0310057X251374691

**Published:** 2025-12-02

**Authors:** Luise Kazda, Kristen M Pickles, Anthony Hull, Alexandra L Barratt, on behalf of the NSW Health Net Zero Clinical Leads Program

**Affiliations:** 1 Healthy Environments and Lives (HEAL) Global Research Centre, Health Research Institute, University of Canberra, Canberra, Australia; 2 Wiser Healthcare Research Collaboration, 4334 Sydney School of Public Health, Faculty of Medicine and Health, The University of Sydney, Sydney, Australia; 3 NSW Health Net Zero Clinical Lead, Bankstown-Lidcombe Hospital, Sydney, Australia

**Keywords:** Desflurane, anaesthesia, greenhouse gas, quality improvement, environment, cost, carbon, sustainability

## Abstract

Desflurane is a potent and expensive greenhouse gas. Reducing its use is a global priority. This anaesthetist-led quality improvement project involved educational, motivational and system-change initiatives implemented in the anaesthesia department of Bankstown-Lidcombe Hospital (BLH) (September 2021–March 2024), with the aim of reducing desflurane consumption. A quasi-experimental interrupted time series design with control site was employed to estimate changes in usage, greenhouse gas emissions and financial cost of anaesthetic agents per 100 surgeries. Prior to intervention, use of desflurane at BLH was stable. During and after intervention, a significant downward trend in desflurane use was observed, reducing by an average of 0.1 units (1 unit = 1 bottle) per month per 100 surgeries from September 2021 onwards (95% confidence interval (CI) –0.21 to –0.01, *P* = 0.035). The intervention, while not directly targeting sevoflurane use, was similarly associated with a downward trend in sevoflurane usage of an average of 0.5 units per month per 100 surgeries from September 2021 onwards (95% CI –189.74 kg to –10.43 kg, *P* = 0.004). No significant changes in use of desflurane or sevoflurane were observed at the control site, although use of both agents declined slightly over the study period. Estimated CO_2_ equivalent (CO_2_e) emissions were reduced by an average of 124.7 kg per month per 100 surgeries from September 2021 onwards (95% CI –223.3 kg to –26.1 kg, *P* = 0.018). Average monthly cost per 100 surgeries at BLH reduced by AU$100.34 per month (95% CI –AU$162.58 to –AU$38.10, *P* = 0.003). There were no changes in CO_2_e emissions or costs at the control site. A clinician-led intervention highlights the importance of creating opportunity and motivation for change amongst staff as well as ongoing education, advocacy and engagement with department and executive to achieve positive environmental and financial outcomes.

## Introduction

Anaesthetic gases account for about 5% of hospital greenhouse gas (GHG) emissions, and up to 50% of perioperative department emissions in high income countries.^1–3^ Desflurane and nitrous oxide (N_2_O) are the worst contributors. The Global Warming Potential (GWP) of desflurane is more than 2500 times that of carbon dioxide (CO_2_) over a 100-year time period (GWP_100_), and even higher (more than 6800) over shorter time frames such as 20 years.^1^ Compared with sevoflurane, a clinically equivalent volatile anaesthetic, desflurane’s GWP_100_ is about 20 times higher, which demonstrates its potent environmental impact. It is also substantially more expensive than available alternatives.^4^ Reduction of its use therefore provides not only planetary health benefits but also significant reductions in hospital financial costs.^5,6^ Hence intervention to decrease use of desflurane is an urgent priority.

In 2022, the European Union announced that desflurane would be banned from 1 January 2026, unless strictly required.^7^ In England, the National Health Service has committed to discontinuing use of desflurane, apart from where necessary clinical indication exists (less than 2% of surgeries),^8^ whilst their Scottish counterpart has already removed desflurane entirely from the supply chain.^9,10^ In Australia, desflurane has been removed from statewide medicine formularies for public hospitals in Western Australia^11^ and New South Wales.^12^ However, desflurane continues to be used in other Australian jurisdictions, in the private sector and globally where its use has not been restricted, remaining a large CO_2_ equivalent (CO_2_e) emissions contributor.

Evidence reviews suggest that multi-component interventions that involve an educational element have a greater likelihood of producing a change in behaviour amongst clinicians than single-component interventions.^13,14^ Furthermore, Zuegge et al have shown that when clinicians receive education about the environmental impact of their clinical activity, they are more motivated to change their clinical behaviour compared with being presented with information about monetary savings.^5^

This study therefore aimed to measure and reduce departmental use of desflurane at a tertiary teaching hospital via an educational, motivational and systems change intervention.

## Methods

### Design and context

This quality improvement project was led by an anaesthetist (AH), NSW Health Net Zero Lead, supported by an academic team of health service researchers (including LK, KMP, ALB) at the University of Sydney as part of the Wiser Healthcare Net Zero partnership.^15^

The intervention and evaluation were conducted in the anaesthetic department of Bankstown-Lidcombe public hospital (BLH), Sydney, Australia, between 2021 and 2024. BLH is a 433-bed teaching and research hospital with eight operating theatres and around 7400 surgeries per year. A second, slightly smaller public hospital (300 beds) in the same local health district and with similar clinical service and surgical specialty provision served as a control site.

Prior to the intervention, BLH staff were varied in their acceptance of the need for emissions reduction, as well as in their knowledge of healthcare’s contribution to carbon emissions, and the impact of global warming on the health of communities and patients. In particular, a lack of detailed understanding about desflurane’s potent GHG effects was observed among clinical staff.

### Intervention

The multi-component intervention was implemented and overseen by AH with support from the district Southwestern Sydney Local Health District (SWSLHD) sustainability officer and committee. A series of educational and system-change initiatives were implemented in the anaesthesia department between September 2021 and March 2024, with the aim of reducing desflurane consumption by educating and encouraging staff to consider the environmental impact of volatile anaesthetics. To describe the components of the intervention, all elements have been mapped to the Capability-Opportunity–Motivation model of behaviour.^16^ The intervention components are described according to the TIDieR checklist for intervention reporting^17^ and a timeline of implementation is supplied (Supplementary material Table S1 and Figure S1 online).

#### Capability

Three in-person group educational sessions (September 2021, May and November 2022) and a trainee-specific teaching session (February 2022) were delivered by AH. The aim of the sessions was to educate anaesthetic consultants, trainees and nursing staff in the department about fundamental sustainability principles in healthcare, the environmental impact of volatile agents, avoiding use of desflurane, alternatives to desflurane and volatile gases, promotion of low flow anaesthesia and propofol-based total intravenous anaesthesia, and the collective responsibility of clinicians for action (see Supplementary Figure S1). Additional information on these topics was disseminated via email, notice board and the Australian and New Zealand College of Anaesthetists bulletin to all department staff. Two grand rounds were delivered to staff at BLH to raise awareness and provide information (February and November 2022). In October 2022, a poster was displayed on a staff notice board in the anaesthetic department showing the carbon footprint of desflurane and methods to reduce volatile anaesthetic emissions, such as substituting with low flow sevoflurane and/or increasing use of intravenous anaesthesia (Supplementary Figure S2). In July 2023 labels detailing the carbon footprint of one bottle of desflurane were attached to each vaporiser (Supplementary Figure S3).

#### Opportunity

Throughout 2022, AH presented at department meetings and to senior executives to increase support for anaesthetic gas reduction initiatives, particularly for the removal of desflurane vaporisers, and for broader emissions reduction activities. As a result, desflurane was removed from all vaporisers (devices that deliver volatile anaesthetics) in December 2022. Desflurane could still be accessed, if required, by a consultant, but doing so involved obtaining the drug from the storeroom. This intervention component aimed to reduce incidental use of desflurane and increase opportunity and motivation to use alternative agents.

#### Motivation

The abovementioned poster included graphs showing trends of volatile and propofol use in the department to provide positive reinforcement and data from other institutions, highlighting their efforts and success of interventions to support reduction of desflurane. A theatre sustainability committee was established at the BLH in November 2022: ‘Greener Bankstown’. The committee comprised over 20 members of variable roles within the operating theatre. The purpose of the committee was to develop, oversee and drive sustainability initiatives in the department.

### Outcomes and measures

The primary outcome was desflurane use. Secondary outcomes were sevoflurane and propofol use, GHG emissions from anaesthetic gases, procurement costs of all anaesthetics (desflurane, sevoflurane, propofol) as well as staff attitudes and behaviour.

#### Anaesthetic agent usage

Desflurane, sevoflurane and propofol monthly usage data (i.e. supplied from pharmacy to clinical areas) was obtained from pharmacy records between January 2021 and March 2024. Propofol usage as anaesthetic was included in the analysis only where it was supplied to the anaesthesia department, operating theatre or recovery to exclude any propofol use beyond anaesthesia, for example, in the intensive care unit or emergency department.

#### Anaesthetic gas GHG emissions

GHG emissions for the two anaesthetic gases in use at BLH were expressed as CO_2_e emissions and calculated using a GWP_100_ of 2540 for desflurane and a GWP_100_ of 130 for sevoflurane^1^ as well as their respective density and volume to determine a carbon footprint of 893 kg/unit for desflurane and 49 kg/unit for sevoflurane.^18^

#### Anaesthetic agent procurement cost

Financial costs were estimated based on averages from pharmacy records between January 2021 and March 2024. A cost of AU$243 per unit of desflurane (240 ml), AU$100 per unit of sevoflurane (250 ml) and AU$2 per vial of propofol (50 ml) was used for all cost calculations.

### Statistical analysis

We used a quasi-experimental interrupted time series design to estimate changes in usage, GHG emissions and financial cost of anaesthetic agents per 100 surgeries attributable to the series of interventions implemented at BLH. We compared observed trends in rates after the beginning of interventions with predicted estimated rates (had interventions not happened) based on baseline trends before the start of the intervention period using segmented autoregression models to statistically estimate aggregate changes in monthly rates. We designed models to include an estimate of a baseline temporal trend (baseline trend) pre-interventions, an estimate of the immediate change due at start of interventions (immediate level change), and an estimate of change to the temporal trend after the beginning of interventions (trend change). At the control site, no interventions occurred, and we simply modelled a baseline trend for visual comparison with our intervention site, estimating a temporal trend for the same time period.

In all analyses, number of surgeries conducted per month was used as the denominator. We calculated the number of all surgeries performed for both sites by combining elective and emergency surgery numbers from two sources. Elective surgeries conducted were reported by quarter by the Bureau of Health Information until December 2023.^19^ We divided the quarterly numbers by 3 to give monthly estimates and used time series analysis to predict values for the first quarter of 2024. Emergency surgeries were reported by financial year up to June 2023 by the Australian Institute of Health and Welfare.^20^ We divided these by 12 to give monthly estimates for emergency surgeries conducted at both sites. We used all available data from July 2011 onwards to forecast a trend and predict values until March 2024. All analyses were conducted in SAS Studio 3.81 (SAS 9.4)

### Ethics

Ethical exemption was granted from SWSLHD Research Ethics Committee SWS46/2023/13.

## Results

### Anaesthetic agent usage

**Figure 1.**
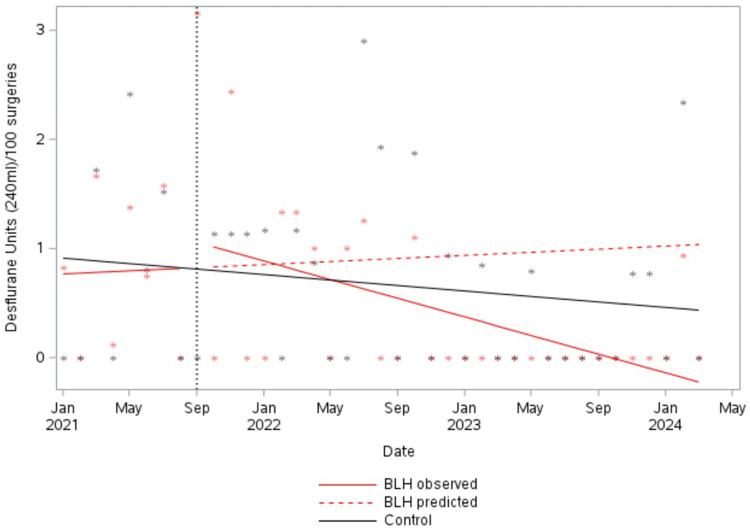
Usage of desflurane per 100 surgeries. BLH: Bankstown-Lidcombe public hospital.

Prior to the start of the multi-component intervention (January to August 2021), desflurane use at BLH was relatively steady (Figure 1). Compared with the pre-intervention trend (Figure 1; BLH predicted), a significant change was detected, with desflurane usage reducing by an average of 0.1 units per month per 100 surgeries from September 2021 onwards (95% confidence interval (CI) −0.21 to −0.01, *P* = 0.035) (Figure 1; BLH observed). Desflurane use at the control site also declined slightly (−0.01 per month, 95% CI −0.04 to 0.01); however, this trend was not significant (*P* = 0.293) (Figure 1; Control).

Similarly to desflurane use, sevoflurane use per 100 surgeries seemed to have been affected by the interventions at BLH. Sevoflurane use at BLH was increasing by 0.4 units per 100 surgeries per month (*P* = 0.011) prior to intervention; however, usage reduced by an average of 0.5 units per month per 100 surgeries from September 2021 onwards (95% CI −0.89 to −0.19, *P* = 0.004) compared with predicted based on the prior trend (Figure S5, Supplementary material). Sevoflurane use at the control site also declined slightly; however, this trend was not significant (–0.04 per month, 95% CI −0.09 to 0.01, *P* = 0.135) (Figure S5, Supplementary material).

There was also a significant change in trend of propofol usage at BLH after the start of interventions, with average units used per month per 100 surgeries dropping by 10.6 (95% CI −14.68 to −6.51, *P*<0.001). At the control site propofol use remained steady over time (*P* = 0.956) (Figure S6, Supplementary material).

### Anaesthetic gas GHG emissions

**Figure 2.**
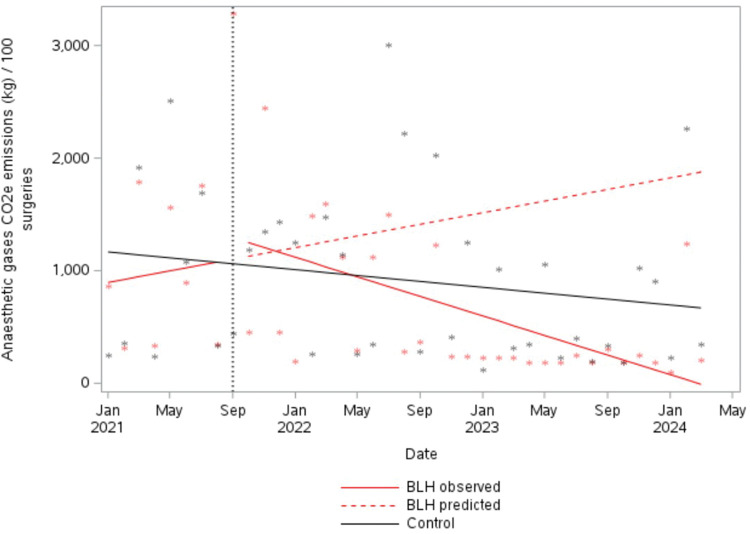
Greenhouse gas emissions from all volatile anaesthetic gases per 100 surgeries. BLH: Bankstown-Lidcombe public hospital; CO_2_e: carbon dioxide equivalent.

GHG emissions from anaesthetic gases declined significantly. Whilst combined CO_2_e emissions per 100 surgeries were trending slightly (insignificantly) upwards at baseline, during the intervention period a significant change in trend was detected, with GHG emissions reducing by an average of 124.5 kg of CO_2_e per month per 100 surgeries from September 2021 onwards (95% CI −223.2 kg to −25.95 kg, *P* = 0.018) (Figure 2).

This decline in GHG emissions from anaesthetic gases was largely driven by the decline in desflurane usage. Emissions from desflurane at BLH declined from around 900 kg CO_2_e per month per 100 surgeries at the start of the interventions to 0 kg of CO_2_e by October 2023 (average reduction of 100.0 kg per month, 95% CI −189.74 kg to −10.43 kg, *P* = 0.035) (Figure S7, Supplementary material). At the control site, average monthly CO_2_e emissions from desflurane use slowly and insignificantly decreased by 11.2 kg per month per 100 surgeries (95% CI −31.8 kg to 9.4 kg, *P* = 0.293) (Figure S7, Supplementary material). An estimated 79,669 kg of CO_2_e emissions (average of 30,840 kg CO_2_e per year) were prevented owing to reduced desflurane usage over the intervention period relative to the baseline period (adjusted for number of surgeries).

### Anaesthetic agent procurement cost

**Figure 3.**
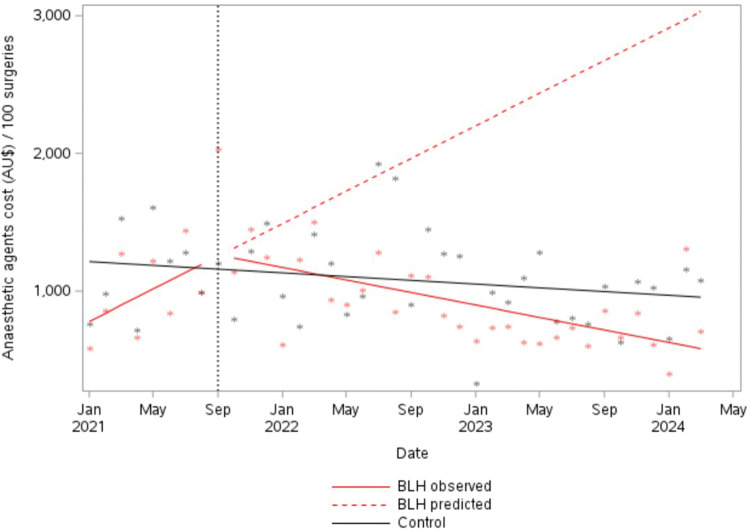
Procurement costs from all anaesthetic agents per 100 surgeries. BLH: Bankstown-Lidcombe public hospital.

Reduced usage of volatile anaesthetics was accompanied by a significant overall reduction in estimated total cost for anaesthetic agents from the beginning of the intervention period. Average monthly cost per 100 surgeries at BLH reduced by AU$100.34 (95% CI –AU$162.58 to −AU$38.10, *P* = 0.003). At the control site, over the entire study period, financial costs also declined slightly; however, this trend was not significant (−AU$6.79 per month, 95% CI −AU$15.98 to AU$2.39, *P* = 0.156) (Figure 3).

Relative to the baseline period and adjusting for surgeries performed, BLH spent an estimated AU$426 less per month on purchasing anaesthetics during the intervention period (average annual saving of AU$5108). Overall, we estimate that BLH made savings of AU$13,196 on purchasing anaesthetic agents during the intervention period (September 2021 to March 2024) compared with the baseline period (adjusted for number of surgeries).

## Discussion

### Principal findings

These educational, motivational and systems change interventions led to a significant reduction in desflurane use at BLH per 100 surgeries per month (down to zero usage coinciding with removal of desflurane from vaporisers), resulting in significant reductions in GHG emissions from anaesthetic gases at the hospital. We estimated that 79,669 kg of CO_2_e emissions (average of 30,840 kg CO_2_e per year) were prevented owing to reduced desflurane usage over the intervention period relative to the baseline period (adjusted for number of surgeries). Moreover, there was an estimated cost saving of AU$13,196 on purchasing anaesthetic agents during the intervention period (September 2021 to March 2024) compared with the baseline period (January to August 2021) (adjusted for number of surgeries).

Somewhat surprisingly, a significant downward trend was also detected for sevoflurane and propofol usage after the intervention. Towards the end of the intervention period (in April 2023), a staff survey was conducted to gauge staff attitudes and practices. By this time, 61% of staff (*n* = 22/36) reported that they did not use desflurane, with more than 70% (*n* = 26/36) indicating they thought it reasonable to remove the desflurane vaporiser from the operating theatre to a storeroom. Fifty-four percent (*n* = 19) of survey respondents used a fresh gas flow rate for desflurane/sevoflurane less than or equal to 0.5l/min, with 43% (*n* = 15) using 0.5–2l/min.

### Findings in relation to other studies

There is global awareness of the need to stop the use of desflurane because of its high GWP,^2,7^ and more than a dozen groups of researchers have conducted interventions to reduce or eliminate its use.^5,21–27^ The interventions studied were most often educational interventions and reminders, with some studies additionally using environmental restructuring (such as removing vaporisers^25,27^) and audit and feedback.^27^ These studies have provided generally weak evidence, as their designs did not include the use of control group. Two randomised controlled trials have been conducted;^28,29^ however, these both tested automated anaesthetic equipment rather than educational or behavioural interventions. Findings across these studies were consistent, demonstrating large reductions in desflurane use (50–95% reductions^5,21–27^) associated with their interventions compared with pre-intervention time periods. In contrast to our study, most reported increases in sevoflurane consumption, with rare exceptions.^21^ Similar to our findings, reductions in costs were commonly reported. To our knowledge all studies of interventions to reduce desflurane have so far been conducted in high-income countries (mostly Europe, the USA and Australia).

### Strengths and limitations

A key strength of our study was the control group to account for coincident temporal changes, as anaesthetists increasingly recognise the need to move away from desflurane.^30^ Providing our findings per 100 surgeries, to allow for any changes in number of surgeries performed over time, together with an interrupted time series analysis, are also important strengths. Finally, and unlike other studies, we have comprehensively reported the components of our intervention using a timeline of intervention components (Figure S4, Supplementary material) and the TIDieR checklist (Table S1, Supplementary material). Our study has limitations as well. A longer pre-intervention time period would have strengthened the design; however, we were unable to obtain reliable purchasing records prior to January 2021. Our control site operates in the same local health district with a small proportion of anaesthetists (approximately 10%) working across both sites. This may have led to some of the educational components of the intervention having an indirect effect on anaesthetic gas usage at the control site. However, our results show no significant reductions in usage at the control site, hence this seems unlikely or only of minor impact. The inclusion of additional intervention and control sites would increase the generalisability of the findings; however, the intervention site is a general metropolitan hospital offering a wide range of surgical services, including those related to cancer, cardiovascular and critical care as well as paediatrics. As such, our findings should be applicable in other similar contexts.

### Implications for practice and policy

At the individual level, a provider’s choice of anaesthetic agent is an important opportunity to mitigate some of healthcare’s GHG emissions at the point of use. While some anaesthetists are aware of the need to reduce atmospheric GHGs, especially over the remainder of this decade, the anaesthetic community are varied in their acceptance of the need for emissions reduction and understanding and awareness of the impact of inhalational anaesthetics on global warming.^2,30^ Additional challenges to the wider use of greener anaesthetic agents have been identified, including provider norms and habits, and time and resource pressures.^30^

Owing to its very large carbon footprint, even infrequent use of desflurane can easily result in it being the major contribution to anaesthetic GHG emissions at a specific hospital site. Therefore, scale-up of interventions to reduce desflurane, and ultimately eliminate it, at other hospitals must remain an important objective to decarbonise the health system. Successful outcomes in this project demonstrate the potential for rapid reduction of anaesthetic GHG emissions and demonstrate the feasibility of eliminating desflurane usage completely. The successful completion of the project can be used to raise awareness and motivate other clinicians towards practice change. Our finding of concomitant reduction in use of sevoflurane was an unexpected co-benefit of intervention that may be of relevance in other contexts.

The success of our intervention relied heavily on the commitment of a local clinical champion (AH) to deliver and sustain the intervention over a prolonged period at the BLH. Support from hospital or health system leadership will be essential to achieve change at other sites and to ensure that the practice change is sustained over time. Local champions—while essential—can only achieve so much because of the effort, time commitment and personal presence required. Support from leadership, combined with numerous local champions, leading to a permanent cultural shift will be needed.

Cessation of desflurane use in anaesthesia remains the ultimate objective. Reduced use by anaesthetists may ultimately alter the business case to the point of borderline viability and eventual withdrawal of the agent. Alternatively, desflurane may be discontinued or banned from sale, as is happening in some jurisdictions.^10–12^ Another related issue is that of ‘desflurane decommissioning’, for expired or unused residual agent. An environmentally sustainable method of achieving this is important.

### Implications for future research

Future studies should aim to use multi-component interventions that go beyond education. The success of our intervention, combining multiple intervention components, is illustrative. Of note, future interventions should be thoroughly described, using the TIDieR checklist to allow replicability and scale-up in other contexts. Equally, future studies should bear in mind the need for a control group to provide findings with low risk of bias that are more likely to be widely taken up in practice.

Although a significant downward trend was detected for sevoflurane and propofol usage after the intervention, we can only speculate as to why this might have occurred. Possible explanations may include greater use of low flow anaesthesia or regional anaesthesia. Investigating the explanation would be of value as the possible explanations could have substantially different environmental impacts.

## Conclusions

A clinician-led quality improvement multi-component intervention has been described and highlights the importance of creating opportunity and motivation for change amongst staff as well as ongoing education, advocacy and engagement with department and executive to achieve positive environmental and financial outcomes.

## Supplemental Material

sj-pdf-1-aic-10.1177_0310057X251374691 - Supplemental material for Reducing use of desflurane in the anaesthetic department: A controlled interrupted time series analysis Supplemental material, sj-pdf-1-aic-10.1177_0310057X251374691 for Reducing use of desflurane in the anaesthetic department: A controlled interrupted time series analysis by Luise Kazda, Kristen M Pickles, Anthony Hull, Alexandra L Barratt; on behalf of the NSW Health Net Zero Clinical Leads Program in Anaesthesia and Intensive Care
