# Supplementary material for: Reducing use of desflurane in the anaesthetic department: A controlled interrupted time series analysis
Source: Anaesth Intensive Care. 2025 Dec 2;54(1):63–71. doi: 10.1177/0310057X251374691 (PMC12779768; doi:10.1177/0310057X251374691)

REDUCING USE OF DESFLURANE IN THE ANAESTHETIC DEPARTMENT - SUPPLEMENTS

Table S1. TIDieR intervention checklist

Figure S1. Educational session presentation

Figure S2. Poster on desflurane emissions

Figure S3. Desflurane vaporiser labels

Figure S4. Timeline of interventions at BLH

Figure S5. Usage of sevoflurane per 100 surgeries

Figure S6. Usage of propofol per 100 surgeries

Figure S7. GHG emissions from desflurane per 100 surgeries

1.

Table S1. TIDieR intervention checklist

**Authors (year)**	Kazda, Pickles, Hull, Barratt (2024)
**Brief name**	Reducing desflurane use in the anaesthetic department
**Recipient**	Anaesthesia department (including anaesthesia consultants, trainees, associated nursing staff)
**Why**	Desflurane is a particularly potent and expensive greenhouse gas; a growing number of hospitals inter/nationally have successfully reduced or eliminated the amount of desflurane used. We observed poor knowledge of the environmental and cost implications of desflurane amongst staff in this department and limited opportunity to facilitate change.
**What (materials)**	-Pinboard outside anaesthetic department providing progress updates (posters with graphs)<br>-Educational poster placed on staff notice board (details of desflurane harm compared with other agents)<br>-Progress update reports via email<br>-Educational materials (pinboard)<br>-Pharmacy labelling on desflurane bottles and vaporisers<br>-Reminder stickers<br>-'A View from Net Zero' article in national anaesthesia bulletin, Autumn 2023
**What (procedures)**	-Staff meetings (integrated into business meetings)<br>-Education and awareness raising/ advocacy (WhatsApp, departmental presentations, trainee induction)<br>-Grand Rounds (education and presentation of results of the initiative)<br>-Formed sustainability committee within department<br>-Staff survey delivered by sustainability committee (Desflurane usage, volatile anaesthesia flows used)<br>-Removed desflurane vaporisers from anaesthetic machines
**Who provided**	An anaesthetist within the anaesthetic department, as part of their net zero lead role, with support from a state-wide sustainability committee<br>No specific training undertaken
**How**	Educational sessions (staff meetings, department meetings, trainee education sessions, nursing sessions, Grand Rounds) were held online and face-to-face<br>Face-to-face staff and departmental meetings occurred monthly with most members of the department present<br>Posters displayed on staff noticeboard (updated occasionally with publications)
**Where**	Anaesthetic Department, Bankstown-Lidcombe Public Hospital, Sydney NSW Australia
**When & how much**	Behavioural and system change interventions progressively implemented between September 2021 and July 2023.<br>Monthly usage and cost data collected for anaesthetic gases from January 2021 to April 2024.

**Tailoring**	Some tailoring of educational materials for trainees
**Modification**	Posters updated occasionally to report on recent research and trends on the topic
**Strategies to improve/maintain intervention fidelity**	Ongoing feedback sought regarding progress, e.g through staff surveys
**Extent of intervention fidelity**	Attendance at educational sessions was recorded by secretary in meeting documents

Figure S1. Educational session presentation

Figure S2. Poster on desflurane emissions

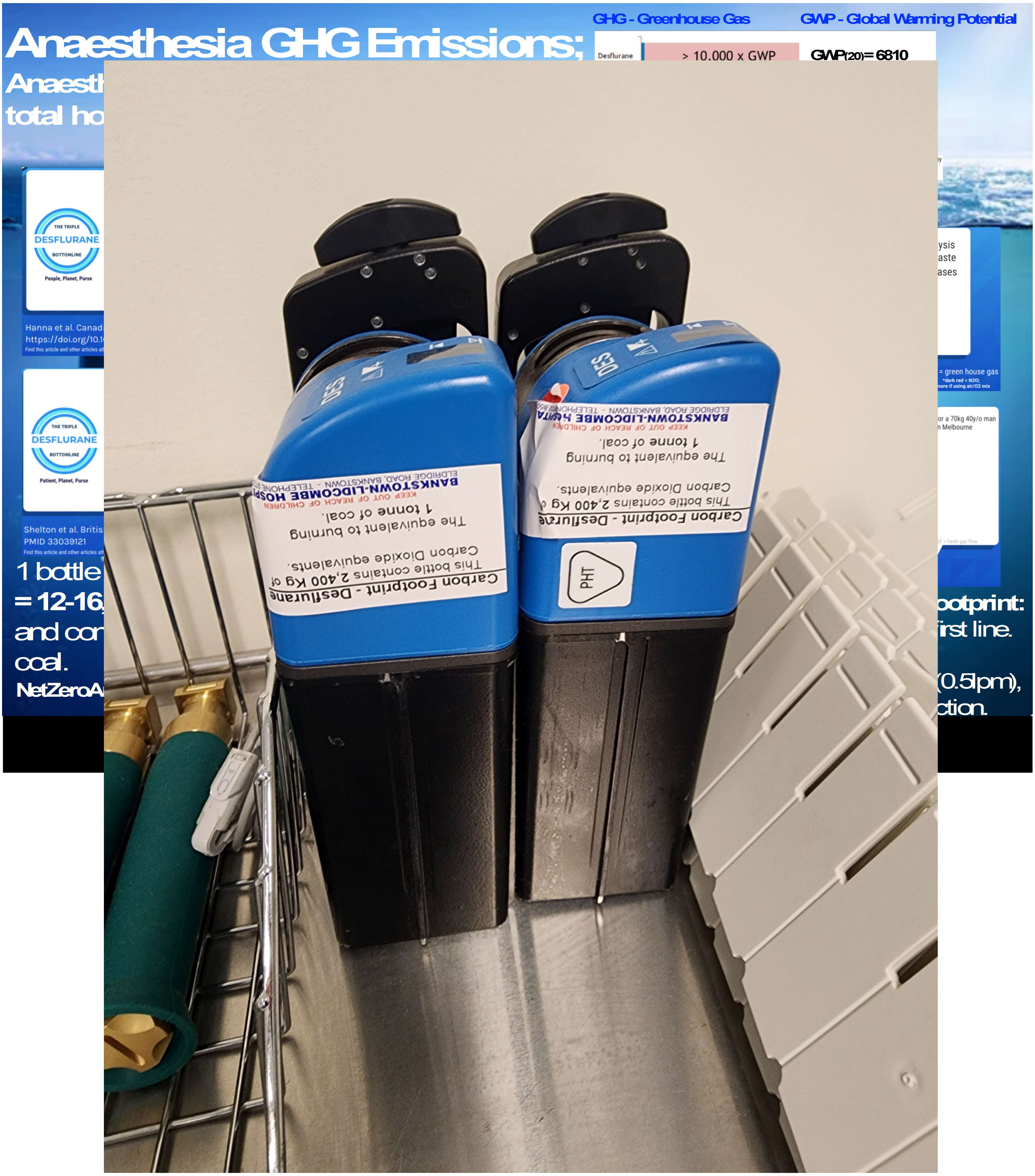

Anaesthesia GHG Emissions;
GHG - Greenhouse Gas
GWP - Global Warming Potential
Desflurane
> 10,000 x GWP
GWP(20)= 6810
THE TRIPLE
DESFLURANE
BOTTOMLINE
People, Planet, Purse
Patient, Planet, Purse
Carbon Footprint - Desflurane
This bottle contains 2,400 Kg of
Carbon Dioxide equivalents.
The equivalent to burning
1 tonne of coal.
BANKSTOWN-LIDCOMBE HOSPITAL
KEEP OUT OF REACH OF CHILDREN
DES
PHT

Figure S4. Timeline of interventions to reduce desflurane use at BLH

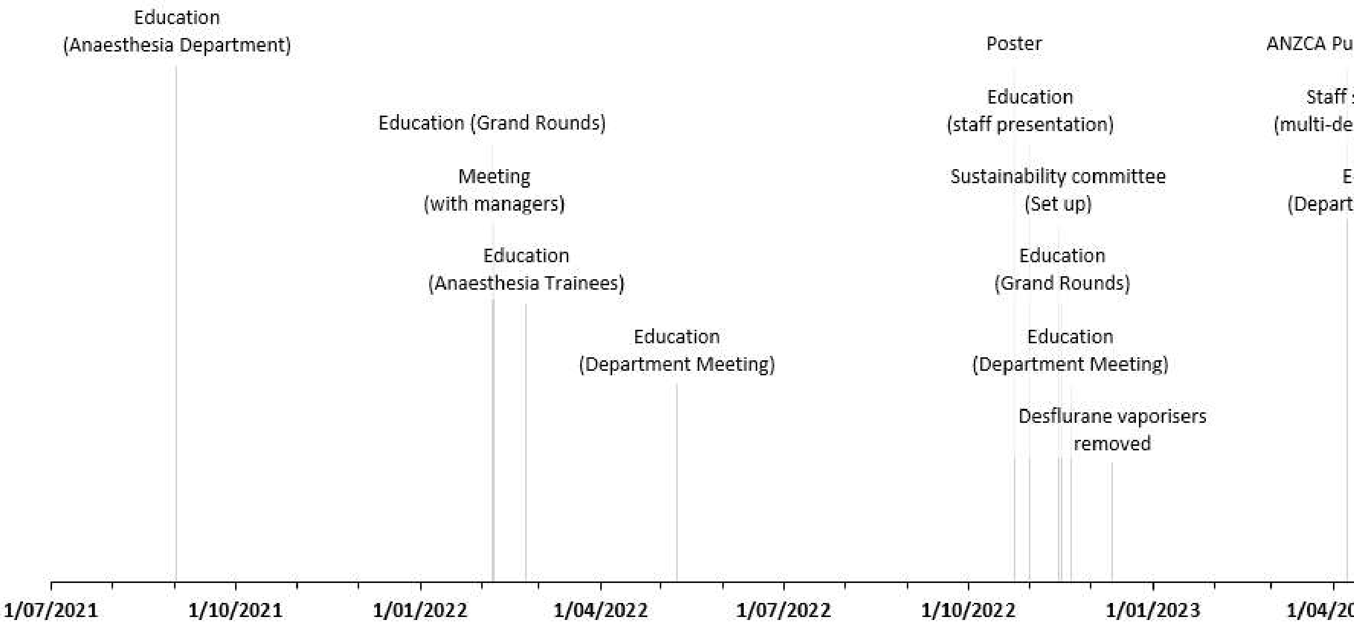

Figure S5. Usage of sevoflurane per 100 surgeries

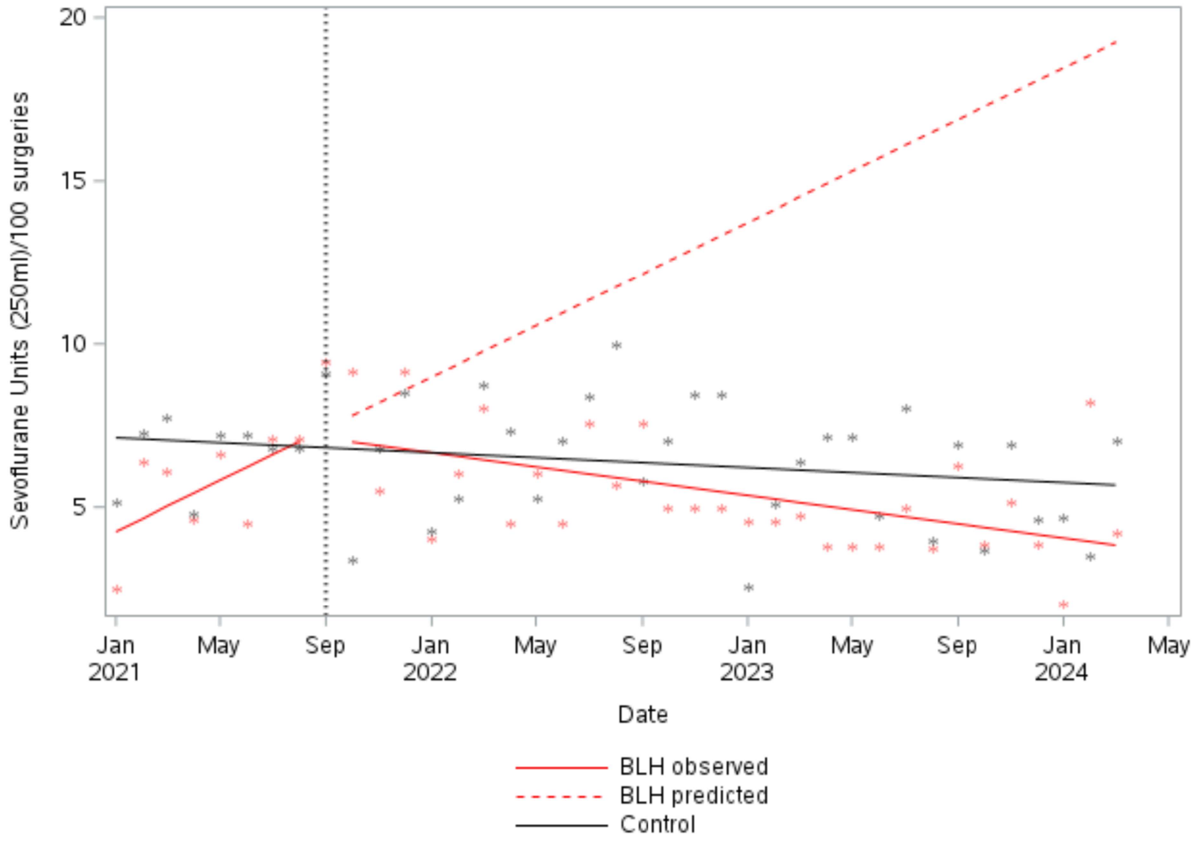

Figure S6. Usage of propofol per 100 surgeries

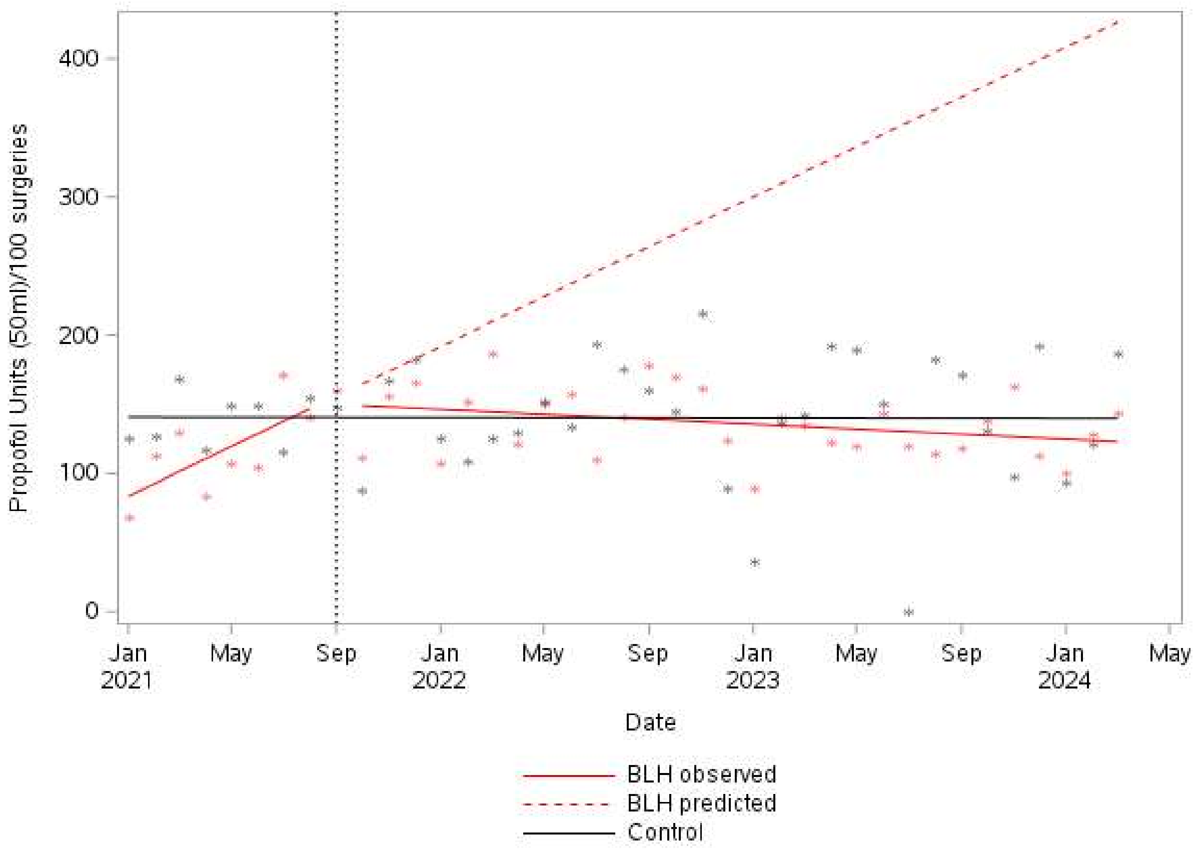

Propofol Units (50ml)/100 surgeries
400
300
200
100
0
Jan 2021
May
Sep
Jan 2022
May
Sep
Jan 2023
May
Sep
Jan 2024
May
Date
BLH observed
BLH predicted
Control

Figure S7. GHG emissions from desflurane per 100 surgeries

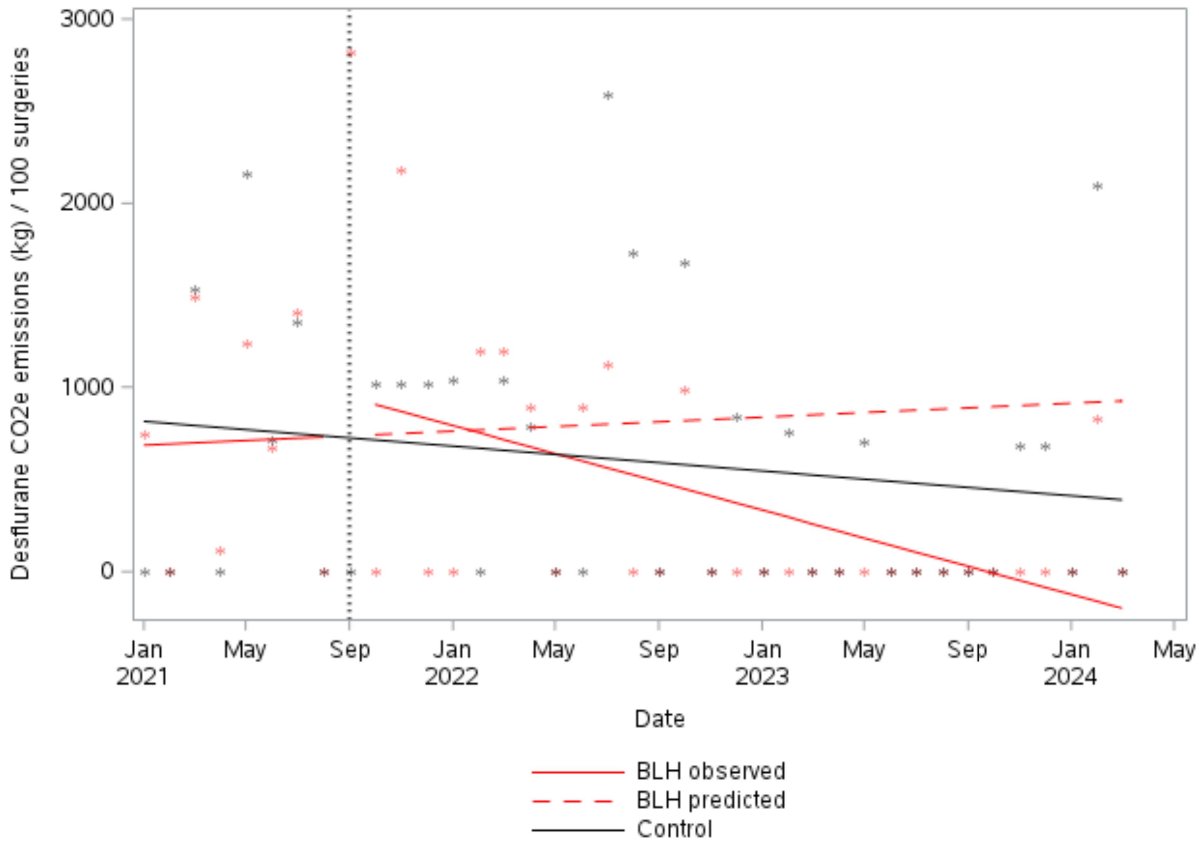

Supplement: sj-pdf-1-aic-10.1177_0310057X251374691 - Supplemental material for Reducing use of desflurane in the anaesthetic department: A controlled interrupted time series analysis [file sj-pdf-1-aic-10.1177_0310057X251374691.pdf]
